# Supplementary material for: Mutations in the KIF21B kinesin gene cause neurodevelopmental disorders through imbalanced canonical motor activity
Source: Nat Commun. 2020 May 15;11:2441. doi: 10.1038/s41467-020-16294-6 (PMC7229210; doi:10.1038/s41467-020-16294-6)
Supplement: Supplementary file 1 — Supplementary Information [file 41467_2020_16294_MOESM1_ESM.pdf]

Supplementary Information

Mutations in the *KIF21B* kinesin gene cause neurodevelopmental disorders through imbalanced canonical motor activity

Asselin et al

Content:

- **Supplementary Note 1:** Clinical features of patients with *KIF21B* variants
- **Supplementary Figure 1:** Patients with *KIF21B* mutations, related to Figure 1.
- **Supplementary Figure 2:** Kif21b expression in mouse developing cortex, related to Figure 2.
- **Supplementary Figure 3:** Expression of *hKIF21B* missense variants induce abnormal neuronal migration, related to Figure 3.
- **Supplementary Figure 4:** KIF21B missense variants induce abnormal neuronal migration through enhanced KIF21B motor activity, related to Figure 4.
- **Supplementary Figure 5:** Overexpression of the p.Gln313Lys *hKIF21B* variant does not impair proliferation in the developing mouse brain, related to Figure 5.
- **Supplementary Figure 6:** p.Ile678Leu hKIF21B variant impedes interhemispheric connectivity through aberrant motor activity, related to Figure 6.
- **Supplementary Figure 7:** Overexpression of p.Ile678Leu hKIF21B variant impairs ipsilateral intracortical axon collaterals formation through a dominant negative effect on motility, related to figure 7.
- **Supplementary Table 1**: List of primary and secondary antibodies used in this work.

Supplementary Note 1: Clinical features of patients with *KIF21B* variants

Patient 1 (NM_001252100.1, c.2032A>C, p.Ile678Leu)

Patient 1 is the second child of healthy, Caucasian, non- consanguineous parents. He was born at full term with normal growth parameters and had an uncomplicated neonatal course. Concerns about development were raised at 18 months when he started walking. He had an uneven gait, stereotypies and no speech. Brain MRI at age two years revealed isolated complete agenesis of the corpus callosum (Fig. 1e). He presented with down-slanting palpebral fissures and downturned corners of the mouth. His first words were at 36 months. He currently has appropriate expressive language despite persistent dysarthria. He was evaluated at six years and nine months (WISC IV) and total IQ was 78 which is consistent with borderline intellectual disability (ID). He has learning disabilities and is therefore cared for in a medico educational institution. He takes methylfenidate due to hyperactivity. Most recent neurological examination was unremarkable. The patient has muscle stiffness, but benefits from physical therapy to ease pain.

Patient 2 (NM_001252100.1, c.937C>A, p.Gln313Lys)

Patient 2 is the child of healthy, non-consanguineous, African American parents. The pregnancy was complicated by intra uterine growth restriction and oligohydramnios. He was born at 38 weeks gestation with height at 49cm (49th percentile), weight at 2.584kg (7th percentile) and head circumference at 32cm (5th percentile). He had a nuchal cord at birth transient cyanosis associated with mild respiratory distress, but was discharged home without complications. He presented with severe developmental delay. He was unable to sit and was non-verbal. Clinical examination at 12 years old showed growth difficulties with weight at 20kg (<1st percentile), height at 139 cm (8th percentile) and microcephaly with head circumference at 48.5cm (<1st percentile, -3.9 SD). He had poor visual fixation with constant tongue thrusting and poor head control. He presented with bilateral ankle tightness, and right wrist contracture.

Patient 3 (NM_001252100.1, c.3001G>A, p.Ala1001Thr)

Patient 3 is a girl of non-consanguineous Dutch parents. Both parents have a reported personal history of mild intellectual disability. The father is a carrier of the variant and present with developmental delay and

learning diffculties. The pregnancy was routine and she was born at full term with normal growth parameters. Her psychomotor development was delayed, she sat at ten months and walked at 24 months. She said her first words at 36 months of age. At age five years, she was able to speak in sentences. She was evaluated at nine years and verbal and performance IQ were 54 and 59 respectively, which is consistent with mild to moderate ID. Upon clinical examination, she had mild dysmoprhic features including epicanthal folds, mild ptosis, and tented upperlip. Her legs were mildly hypertonic. The brain MRI did not show any structural abnormalities.

Patient 4 (NM_001252100.1, c.2959_2962dup, p.Asn988Serfs*4)

Patient 4 was born to non-consanguineous parents. Mother had a seizure disorder and she took Topiramate during the pregnancy. Antenatal ultrasound was positive for the fetus measuring small for gestational age. He was born at 38 weeks gestation by cesarean section secondary to repeat maternal seizures. Neonatal mensurations confirmed hypotrophy with birth height at 43 cm (<1st percentile) and birth weight at 2633 g (8th percentile). He had feeding difficulties in the neonatal period requiring an NG tube. Problems persisted and he received a G-tube at 18 months. Currently, he takes mainly by mouth and uses the G-tube for medications. He has a history of moderate to severe constipation. At 37 months of age, he had a developmental quotient of 97 consistent with a history of mild global developmental delays. He was diagnosed with right Duane syndrome and has central sleep apnea requiring C pap after adenoidectomy. He had a chromosome microarray (CMA) that was non-diagnostic; it showed he was a carrier for Poretti-Boltshauser syndrome [OMIM: 615960] due to a single heterozygous LAMA1 deletion and a gain on 22q11.23 with no clinical consequence associated. He has also had normal mitochondrial sequencing.

Supplementary Figure 1: Patients with *KIF21B* variants, related to Figure 1.

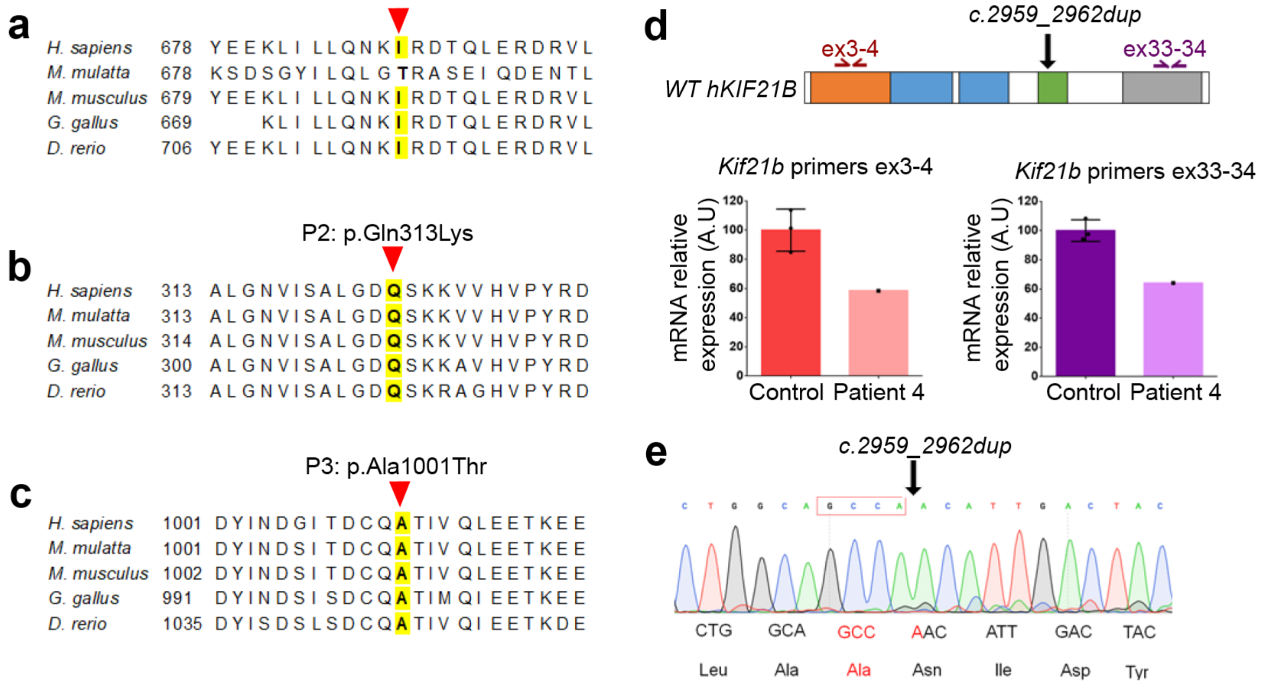

f

Patient No.	Sequence changes in *KIF21B gene* (NM_001252100.1)	Align GVGD	SIFT	MutationTaster	Polyphen-2	CADD
1	c.2032A>C, p.Ile678Leu	Class C0 (GV: 241.08 - GD: 4.86)	Tolerated (score: 0.05)	**Disease causing** (p-value: 1)	**Probably damaging** with a score of 0.960 (sensitivity: 0.78; specificity: 0.95)	25.4
2	c.937C>A, p.Gln313Lys	Class C0 (GV: 223.30 - GD: 36.80)	**Deleterious** (score: 0)	**Disease causing** (p-value: 1)	**Probably damaging** with a score of 0.985 (sensitivity: 0.74; specificity: 0.96)	38
3	c.3001G>A, p.Ala1001Thr	Class C55 (GV: 0.00 - GD: 58.02)	**Deleterious** (score: 0)	**Disease causing** (p-value: 1)	**Probably damaging** with a score of 0.997 (sensitivity: 0.41; specificity: 0.98)	29.1

(**a-c**) Alignment of the KIF21B protein across several species (human, macaque, mouse, fish, chicken, zebrafish) shows the conservation of the mutated amino acid residue (red arrow head) in patients. (**d**) RT-qPCR analyses showing a decrease of *KIF21B* mRNA expression in Patient 4 (carrying a duplication c.2959_2962dup, p.Asn988SerfsX4). Three male individuals were used as control and each dot represent one independent measure. Data are represented as means ± S.E.M. Position of the primers used for qPCR are shown in the upper panel. (**e**) Chromatogram showing the results of Sanger sequencing of RNA isolated from Patient 4's blood. (**f**) *In silico* prediction of sequence changes identified in patients, based on human *KIF21B* transcript variant 1 (RefSeq: NM_001252100.1). Source data are provided in the Source Data file.

Supplementary Figure 2: Kif21b expression in mouse developing cortex, related to Figure 2.

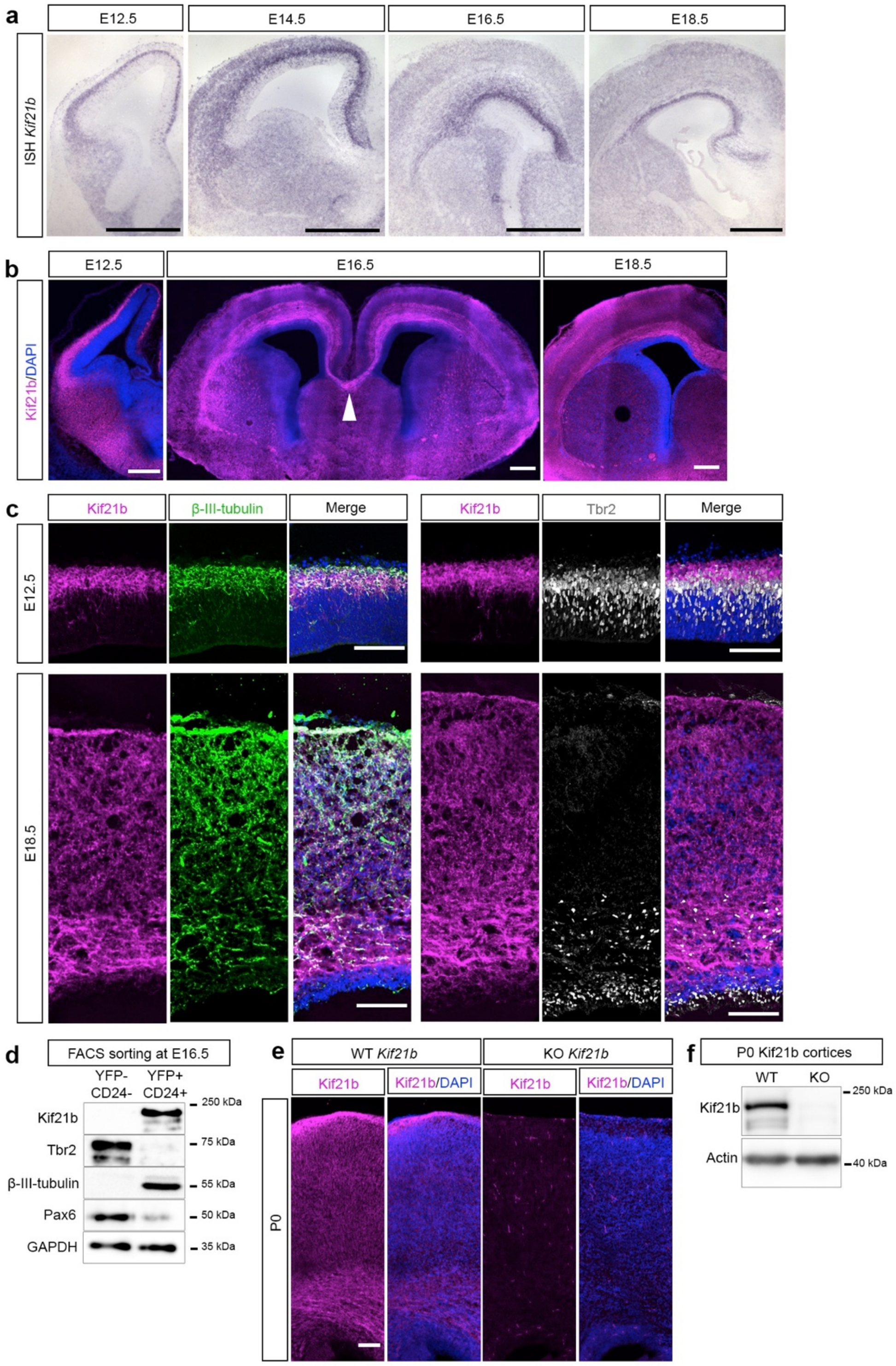

(**a**) *In situ* hybridization of mouse brain sections (from E12.5 to E18.5) showing m*Kif21b* transcripts distribution. (**b-c**) E12.5, E16.5 and E18.5 mouse brains section immunolabelled for Kif21b (magenta), β-III-tubulin (neuronal marker, green) and Tbr2 (intermediate progenitor marker, light grey) showing restricted expression of Kif21b in post-mitotic neurons and enrichment in the axon-rich zone (**b**, white arrowhead). (**d**) Western blot of extracts from progenitors (YFP-; CD24-, expressing Pax6 and Tbr2 transcription factors) and neurons (YFP+; CD24+, expressing β-III-tubulin) isolated by FACS from Rosa26-loxSTOP-YFP; NEX$^{CRE/+}$ E16.5 mouse cortices showing that Kif21b expression is restricted to neuronal population (n=3 independent experiments). GAPDH was used as a loading control. (**e**) Immunolabelling of Kif21b on P0 wild-type (WT) or Kif21b knock-out (KO) mouse brain confirm the specificity of the Kif21b staining. (**f**) Western blot of extracts from WT or Kif21b KO cortices showing the absence of Kif21b expression in KO brain (n=3 brains per genotype). Scale bars, (**a**) 500µm, (**b**) 250µm and (**c, e**) 100µm. (**b, c, e**) Nuclei are stained with DAPI. Source data are provided in the Source Data file.

Supplementary Figure 3: Expression of *hKIF21B* missense variants induce abnormal neuronal migration, related to Figure 3.

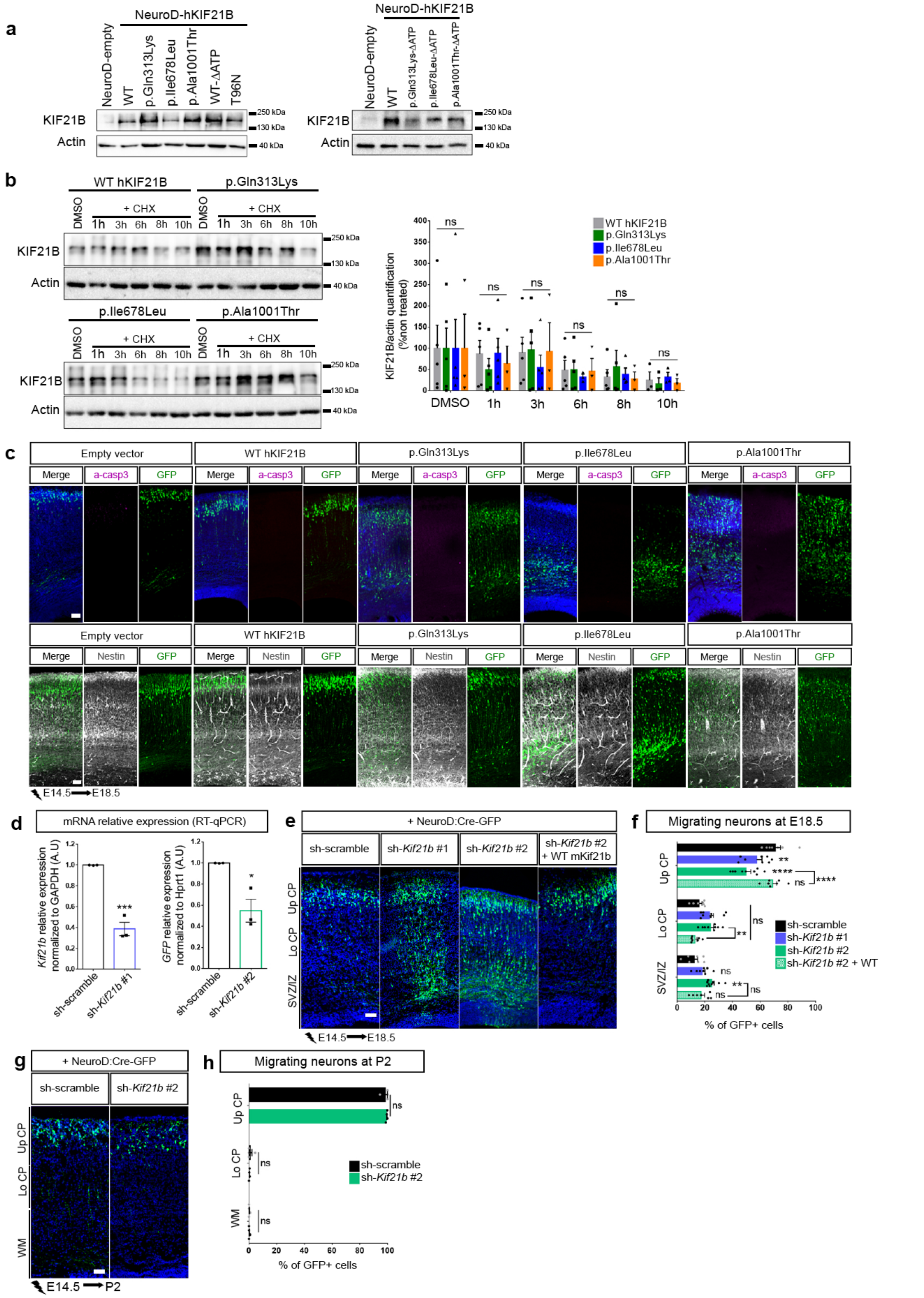

(**a**) Western blot of extract from N2A cells transfected with the indicated NeuroD-IRES-GFP constructs showing similar expression of both WT and mutant hKIF21B protein. Actin was used as a loading control. (**b**) Cycloheximide (CHX, 10µg/ml) treatment for the indicated duration show similar half-life of WT and mutant hKIF21B proteins. Data (means ± S.E.M) were analyzed by two-way ANOVA, Bonferroni's multiple comparisons test, ns, non-significant. Experiments were repeated five times for WT, p.Gln313Lys and p.Ile678Leu conditions and three times for pAla1001Thr condition. (**c,** upper panel) Activated caspase3 (a-casp3, magenta)- or (lower panel) nestin (grey)-immunolabelling of E18.5 mouse brain coronal sections electroporated at E14.5 with the indicated NeuroD-hKIF21B IRES-GFP constructs, showing no apoptosis defects and no glia scaffold impairment. GFP positive electroporated cells are depicted in green. Nuclei are stained with DAPI. (**d**) RT-qPCR of HEK293T cells transfected with the indicated constructs showing *Kif21b* knock-down efficiency. Data (means ± S.E.M) from 3 independents experiments were analyzed by unpaired two-tailed Student t-test, *P < 0.05; ***P < 0.001. (**e, g**) Coronal sections of (**e**) E18.5 or (**g**) P2 mouse brains electroporated at E14.5 with NeuroD:Cre-GFP together with either Cre inducible shRNA-*Kif21b* #1 or #2 or sh-scramble sequence. Rescue experiments were done by co-expressing NeuroD:Cre-GFP, inducible shRNA-*Kif21b* #2 together with WT-hKIF21B at 1 µg/µl. GFP positive electroporated cells are depicted in green. Nuclei are stained with DAPI. (**f, h**) Histograms (means ± S.E.M) showing the distribution of GFP-positive neurons in different regions (Up CP, Upper cortical plate; Lo CP, Lower cortical plate; IZ, intermediate zone; SVZ, subventricular zone) for all conditions as indicated. Significance was calculated by two-way ANOVA (Bonferroni's multiple comparisons test). Number of embryos analyzed: (**f**) sh-scramble, n=7; sh-*Kif21b* #1, n=8; sh-*Kif21b* #2, n=9; sh-*Kif21b* #2 + WT, n=7; (**h**) sh-scramble, n=4; sh-*Kif21b* #2, n=4.; ns, non-significant; **P < 0.005; ****P < 0.0001. Scale bars (**c**, **e, g**), 50 µm. Source data are provided in the Source Data file.

Supplementary Figure 4: hKIF21B missense variants induce abnormal neuronal migration through enhanced KIF21B motor activity, related to Figure 4.

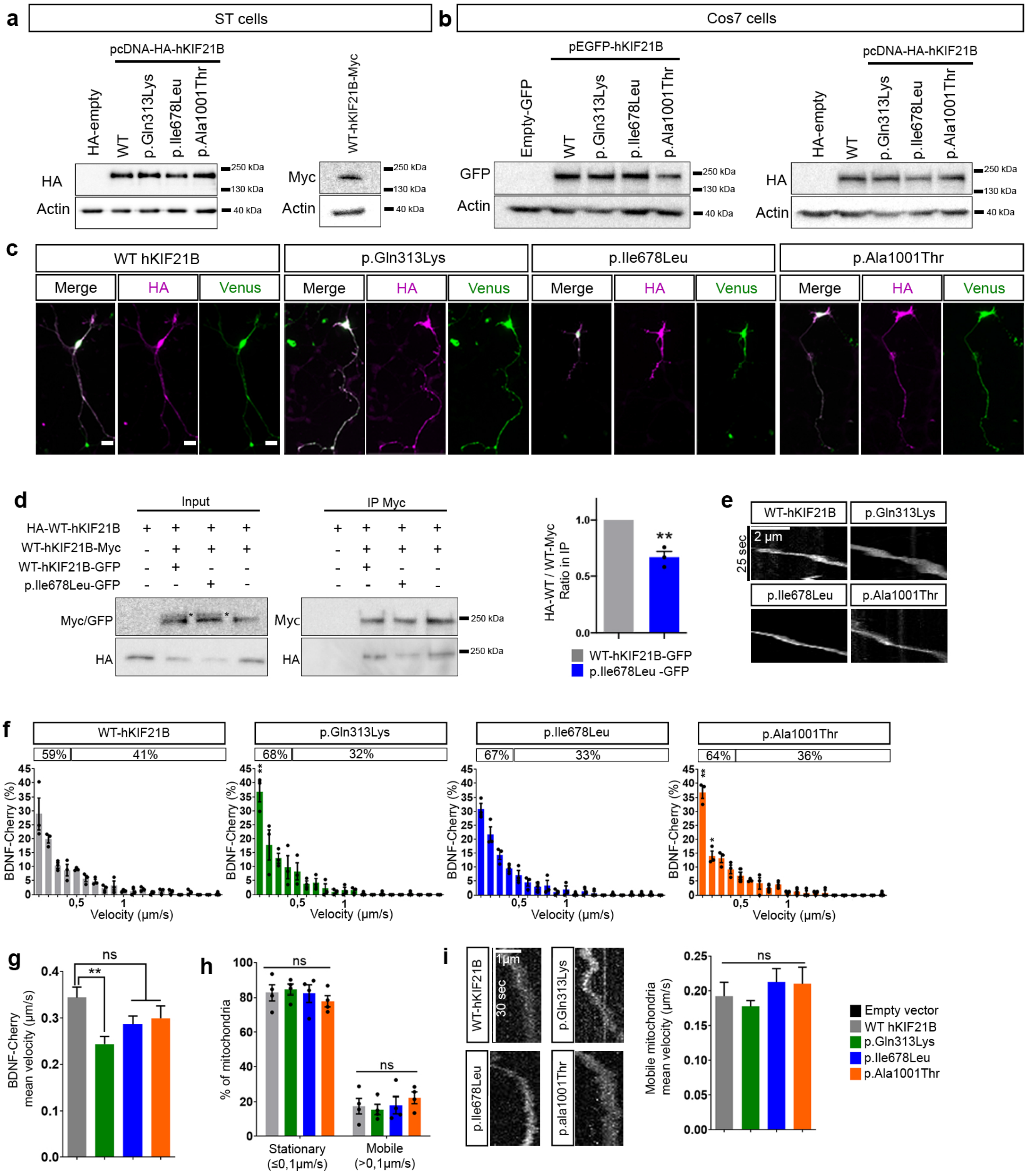

(**a, b**) Western blot of extract from (**a**) ST cells transfected with the indicated HA-tagged or Myc-tagged constructs or (**b**) Cos7 cells transfected with the indicated GFP-tagged constructs or HA-tagged constructs, showing similar expression of both WT and mutant hKIF21B protein. Actin was used as a loading control. (**c**) HA-immunolabelling of primary cortical neurons transfected with WT or mutant HA-tagged hKIF21B cDNA constructs together with pCAGGs-mVenus showing distribution of KIF21B (HA, magenta) in soma, dendrites and axons at DIV2. Scale bars, 20 µm. (**d**) WT hKIF21B immunoprecipitation (Myc beads, IP Myc) experiments in HEK293T cells transfected with the indicated HA-tagged, Myc-Tagged and GFP-tagged constructs showing that the p.Ile678Leu variant is competing with the wild type protein to form KIF21B homodimer. Whole cell lysates are shown as input. The asterisk shows the band corresponding to the GFP-tagged protein. Histograms (means ± S.E.M) represent the quantitative assessment of the band intensities of the coimmunoprecipitated

versus immunoprecipitated WT proteins from 3 independent experiments (unpaired two-tailed Student t-test, **P < 0.005). (**e-i**) Cos7 cells were co-transfected with BDNF-Cherry or Mito-RFP plasmids together with WT or mutant HA-tagged hKIF21B constructs and analyzed by videomicroscopy. Kymographs illustrate the motility of BDNF vesicles (**e**) or mitochondria (**i**) in time (y, sec) and space (x, µm). Histograms (means ± S.E.M) represent the distribution of BDNF particles velocities (**f**), the percentage of stationary mitochondria (velocity ≤0,1µm/s) *versus* mobile mitochondria (velocity >0,1µm/s) (**h**) and the mean velocities of (**g**) BDNF particles and (**i**) mobile mitochondria. Data were analyzed (**f, h**) by two-way ANOVA or (**g, i**) by one-way ANOVA (Bonferroni's multiple comparisons test). (**f**, **g**) Total number of BDNF particles analysed in 10 to 19 cells: WT, n=322; p.Gln313Lys, n=255; p.Ile678Leu, n=307; pAla1001Thr, n=250. Experiments were repeated three times for all conditions. (**h**, **i**) Total number of mitochondria analysed in 19 to 31 cells: WT, n=177; p.Gln313Lys, n=256; p.Ile678Leu, n=189; pAla1001Thr, n=173. Experiments were repeated four times for all conditions. ns, non-significant; *P < 0.05; **P < 0.005. Source data are provided in the Source Data file.

Supplementary Figure 5: Overexpression of the p.Gln313Lys *hKIF21B* variant does not impair proliferation in the developing mouse brain, related to Figure 5.

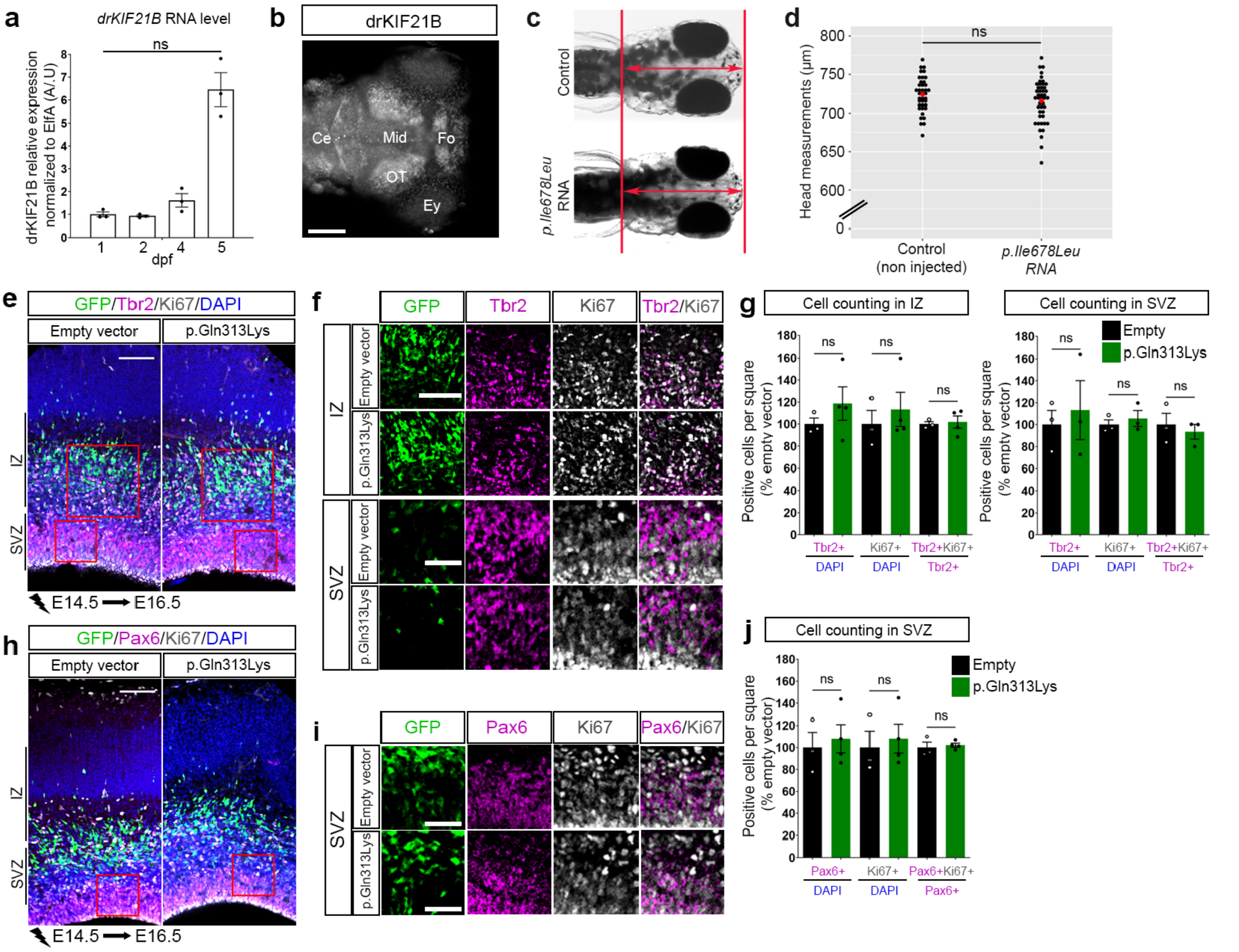

(**a**) qRT-PCR analyses show expression of *drKif21b* transcripts in developing zebrafish larvaes at 1, 2, 4 and 5 days post-fertilization (dpf). Data (means ± S.E.M) from 3 technical replicates per stage were analyzed by one-way ANOVA (Bonferroni's multiple comparisons test). ns, non-significant. (**b**) Kif21b-immunolabelling (grey) of whole zebrafish larvae show Kif21b enrichment in different region of the brain (OT, optic tectum; Ey, eye; Fo, forebrain; Mid, midbrain; Ce, cerebellum). (**c**) Dorsal view of representative control zebrafish larvae (non-injected) or injected with 100 pg of p.Ile678Leu *hKIF21B* RNAs at 5 dpf. Double arrow indicates the distance between the forebrain and hindbrain, a measure used as a proxy for head size. (**d**) Dot plot of the head measurements (red double arrow) of control and RNA-injected larvae at 5dpf. Red diamond corresponds to the mean of the batch measured. Significance was calculated by unpaired two-tailed Student t-test comparisons between control and RNA-injected larvae. Number of embryos analyzed for this specific batch: control, n=45; pIle678Leu, n=39. Experiments were repeated three times for each condition. Total Number of embryos analyzed: control, n=107; pIle678Leu-injected embryos, n=105. ns, non-significant. (**e, f, h, i**) Tbr2 (magenta) (**e**, **f**) of Pax6 (magenta) (**h**, **i**) and Ki67 (grey) double immunolabeling of coronal sections of E16.5 mouse cortices electroporated at E14.5 with NeuroD-IRES-GFP empty vector or NeuroD-hKIF21B-p.Gln313Lys. GFP positive electroporated cells are depicted in green. Nuclei are stained with DAPI. (**g**, **j**) Analysis (means ± S.E.M) of the percentage of Tbr2-positive cells, Pax6-positive cells, Ki67-positive cells or cells double positive for Tbr2 (**g**) or Pax6 (**i**) and Ki67 in the intermediate zone (IZ) or sub-ventricular zone (SVZ) in a fixed-size square represented in (**e**) and (**h**). Data were analyzed by unpaired two-tailed Student t-test. Number of embryos analyzed for (**g**) IZ countings, WT, n=4; p.Gln313Lys, n=3; and for (**g**, **j**) SVZ countings, WT, n=3; p.Gln313Lys, n=3. ns, non-significant. Scale bars, (**b**) 10 µm; (**e, h**) 100 µm (**f**, upper panel) 100 µm, (**f**, lower panel and **i**) 50 µm. Source data are provided in the Source Data file.

Supplementary Figure 6: p.Ile678Leu hKIF21B variant impedes interhemispheric connectivity through aberrant motor activity, related to Figure 6.

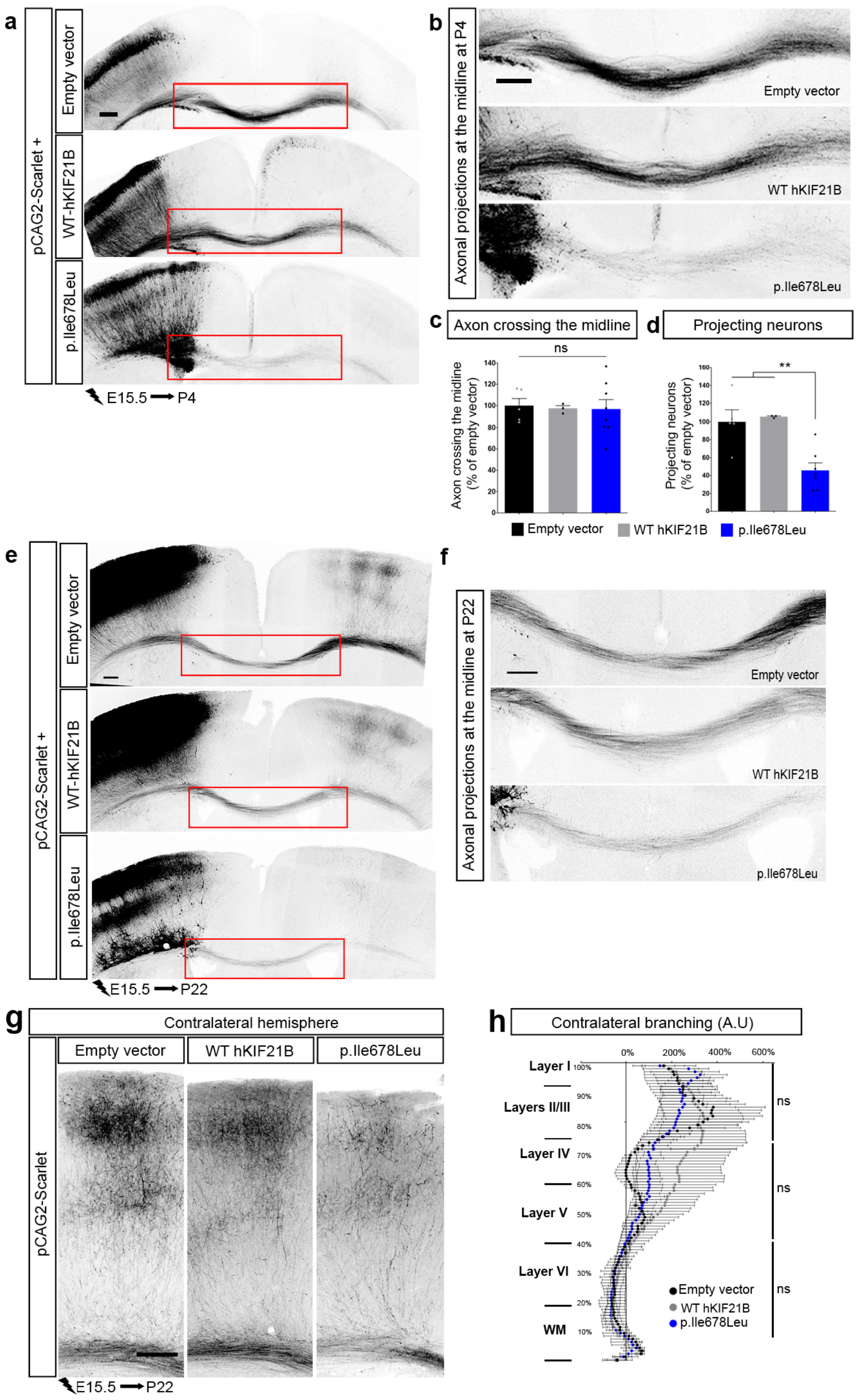

(**a, e**) Coronal sections of (**a**) P4 or (**e**) P22 mouse brains after IUE with pCAG2-Scarlet and a NeuroD-IRES-GFP empty vector or WT or p.Ile678Leu NeuroD-hKIF21B-IRES-GFP constructs at E15.5. (**b, f**) Close-up views of the red boxed area in (**a**) or (**e**) showing impaired axonal interhemispheric connectivity upon expression of the p.Ile678Leu variant both at P4 and P22. (**c-d**) Histograms (means ± S.E.M) presenting the quantification of (**c**) the percentage of axon crossing the midline and (**d**) the percentage of projecting neurons for all conditions tested as indicated at P4. Data were analyzed by one-way ANOVA (Bonferroni's multiple comparisons test). Number of pups analyzed: (**c**) empty vector, n=5; WT, n=3; p.Ile678Leu, n=8; (**d**) empty vector, n=5; WT, n=3; p.Ile678Leu, n=7. ns, non-significant, **P < 0.005. (**g**) Contralateral side of P22 mouse brain coronal sections electroporated with pCAG2-Scarlet and a NeuroD-IRES-GFP empty vector or WT or p.Ile678Leu NeuroD-IRES-GFP constructs at E15.5 showing no terminal branching defects for all conditions tested. (**h**) Quantification of normalized pScarlet fluorescence along the radial axis of the cortical wall in the contralateral cortex. Data (means ± S.E.M) were analyzed by two-way ANOVA (Bonferroni's multiple comparisons test). Number of animals analyzed: empty vector, n=6; p.Ile678Leu, n=4; ns, non-significant. Scale bars (**a**, **b**, **e**, **f** and **g**), 250 µm. Source data are provided in the Source Data file.

Supplementary Figure 7: Overexpression of p.Ile678Leu hKIF21B variant impairs ipsilateral intracortical axon collaterals formation through a dominant negative effect on motility, related to figure 7.

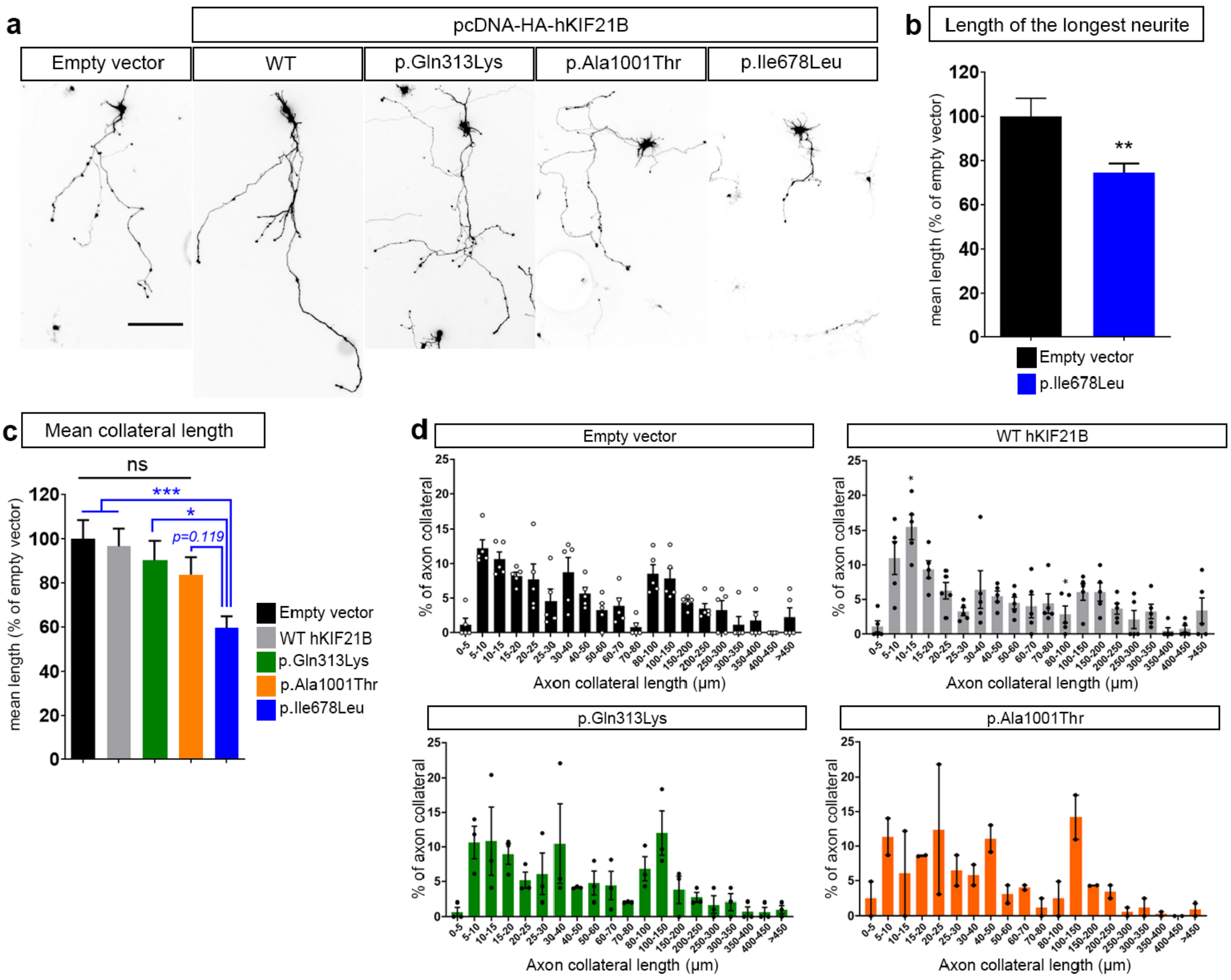

(**a**) Representative DIV5 cortical neurons magnetofected at DIV2 with pCAG2-Scarlet together with control empty pcDNA-HA or WT, p.Gln313Lys, pAla1001Thr or p.Ile678Leu pcDNA-HA-hKIF21B constructs. Scale bar, 150 µm. (**b,c**) Quantification of (**b**) the longest neurite length (axon) or (**c**) the mean of collaterals length at DIV5 after overexpression of the indicated constructs. (**d**) Distribution of axon collateral branches length at DIV5 after overexpression of the indicated constructs. (**b-d**) Data (means ± S.E.M) of (**b**) the longest neurite length, (**c**) the mean of collateral length or (**d**) the distribution of axon collaterals length were analyzed by (**b**) unpaired two-tailed Student t-test, (**c**) one-way ANOVA or (**d**) two-ways ANOVA (Bonferroni's multiple comparisons test). (**b**) Number of cells analyzed: empty vector, n=66; p.Ile678Leu, n=51; from five independent experiments. (**c**, **d**) Number of collaterals analyzed: empty vector, n=265; WT, n=322; p.Gln313Lys, n=192; p.Ile678Leu, n=385; p.Ala1001Thr, n=187; in 51, 62, 66, 37 or 30 cells respectively, from five (empty vector, WT, p.Ile678Leu), three (p.Gln313Lys) or two (p.Ala1001Thr) independent experiments. ns, non-significant; *P < 0.05; **P < 0.005; ***P < 0.001. Source data are provided in the Source Data file.

Supplementary Table 1: List of primary and secondary antibodies used in this work.

Antibody	Host	Dilution	Used for	Compagny	reference
β-III-tubulin	Mouse	1/200	IHC, WB	Eurogentec	MMS-435P-0100
Caspase-3	Rabbit	1/100	IHC	R and D system	AF835
Cux1	Rabbit	1/200	IHC	Proteintech	HPA003317
GFP	Goat	1/500	IHC, WB	Abcam	ab6673
GFP	Chicken	1/500	IHC	Abcam	GFP-1020
HA	Rat	1/1000	IF, WB	Sigma-Aldrich	11867423001
Myc-Tag	Rabbit	1/1500	IF, WB	Cell Signalling	71D10
KIF21B	Rabbit	1/200	IHC, IF, WB	Sigma-Aldrich	HPA027274
KIF21B	Rabbit	1/500	IF (zebrafish staining)	Abcam	ab135410
Pax6	Rabbit	1/500	IHC	Biolegends	901301
Tau	Mouse	1/1000	IF	Millipore	MAB3420
Tbr2	Rat	1/250	IHC, WB	EBiosciences	14-4875-80
Actin coupled HRP	Mouse	1/100 000	WB	Sigma-Aldrich	A3854
Goat-mouse-HRP	Mouse	1/10 000	WB	ThermoFisher Sc.	G-21040
Goat-rabbit-HRP	Rabbit	1/10 000	WB	ThermoFisher Sc.	G-21234
Goat-rat-HRP	Rat	1/10 000	WB	ThermoFisher Sc.	62-9520
Donkey-goat-488	Goat	1/1000	IF	ThermoFisher Sc.	A-11055
Donkey-mouse-488	Mouse	1/1000	IF	ThermoFisher Sc.	A-21202
Donkey-mouse-555	Mouse	1/1000	IF	ThermoFisher Sc.	A-31570
Donkey-rabbit-488	Rabbit	1/1000	IF	ThermoFisher Sc.	R-37118
Donkey-rabbit-555	Rabbit	1/1000	IF	ThermoFisher Sc.	A-31572
Donkey-rat-488	Rat	1/1000	IF	ThermoFisher Sc.	A-21208
Ki67 coupled-570	Rat	1/500	IF	eBioscience	41 5698 80

IHC, immunohistochemistry, IF, immunofluorescence; WB, western blot.
