# Supplementary material for: Mutations in the KIF21B kinesin gene cause neurodevelopmental disorders through imbalanced canonical motor activity
Source: Nat Commun. 2020 May 15;11:2441. doi: 10.1038/s41467-020-16294-6 (PMC7229210; doi:10.1038/s41467-020-16294-6)
Supplement: Supplementary file 3 — Description of Additional Supplementary Files [file 41467_2020_16294_MOESM3_ESM.pdf]

Description of Additional Supplementary Files

File Name: Supplementary Data 1
Description: A detailed description of statistics behind all graphical representation of data.
