# Supplementary material for: Mutations in the KIF21B kinesin gene cause neurodevelopmental disorders through imbalanced canonical motor activity
Source: Nat Commun. 2020 May 15;11:2441. doi: 10.1038/s41467-020-16294-6 (PMC7229210; doi:10.1038/s41467-020-16294-6)
Supplement: Supplementary file 5 — Reporting Summary [file 41467_2020_16294_MOESM5_ESM.pdf]

nature research

Corresponding author(s): Juliette D. Godin

Last updated by author(s): Apr 22, 2020

Reporting Summary

Nature Research wishes to improve the reproducibility of the work that we publish. This form provides structure for consistency and transparency in reporting. For further information on Nature Research policies, see Authors & Referees and the Editorial Policy Checklist.

Statistics

For all statistical analyses, confirm that the following items are present in the figure legend, table legend, main text, or Methods section.

n/a	Confirmed	
☐	☒	The exact sample size (n) for each experimental group/condition, given as a discrete number and unit of measurement
☐	☒	A statement on whether measurements were taken from distinct samples or whether the same sample was measured repeatedly
☐	☒	The statistical test(s) used AND whether they are one- or two-sided<br>*Only common tests should be described solely by name; describe more complex techniques in the Methods section.*
☒	☐	A description of all covariates tested
☐	☒	A description of any assumptions or corrections, such as tests of normality and adjustment for multiple comparisons
☐	☒	A full description of the statistical parameters including central tendency (e.g. means) or other basic estimates (e.g. regression coefficient) AND variation (e.g. standard deviation) or associated estimates of uncertainty (e.g. confidence intervals)
☐	☒	For null hypothesis testing, the test statistic (e.g. F, t, r) with confidence intervals, effect sizes, degrees of freedom and P value noted<br>*Give P values as exact values whenever suitable.*
☒	☐	For Bayesian analysis, information on the choice of priors and Markov chain Monte Carlo settings
☒	☐	For hierarchical and complex designs, identification of the appropriate level for tests and full reporting of outcomes
☒	☐	Estimates of effect sizes (e.g. Cohen's d, Pearson's r), indicating how they were calculated

Our web collection on statistics for biologists contains articles on many of the points above.

Software and code

Policy information about availability of computer code

Data collection	Epifluorescence and confocal images: Leica Las X software v3.7; Videomicroscopy: Metamorph software v7.6; Macroscope images: CoolSnap v1.2
Data analysis	The bioinformatic analyses was conducted by Polyweb using BWA 0.7.12, picard-tools-1.121, GenomeAnalysisTK-2014.3- 17-g0583013, SNPEff-4.2. Images analysis were performed using ImageJ/FIJI (64-bit Java 1.8.0_112) and plugins Manual Tracking (https://imagej.nih.gov/ij/plugins/track/track.html), Simple Neurite Tracer (https://imagej.net/Simple_Neurite_Tracer), KymoTool_Box (v1.01 https://github.com/fabricecordelieres/IJ-Plugin_KymoToolBox), ProfilePlot (https://imagej.nih.gov/ij/docs/guide/146-30.html#sub:Plot-Profile-[k])). Statistical analysis were performed using Prism (v6, GraphPad).

For manuscripts utilizing custom algorithms or software that are central to the research but not yet described in published literature, software must be made available to editors/reviewers. We strongly encourage code deposition in a community repository (e.g. GitHub). See the Nature Research guidelines for submitting code & software for further information.

Data

Policy information about availability of data

All manuscripts must include a data availability statement. This statement should provide the following information, where applicable:

- Accession codes, unique identifiers, or web links for publicly available datasets
- A list of figures that have associated raw data
- A description of any restrictions on data availability

The following databases and in silico software were used in the study: Human Gene Mutation Databases (http://www.hgmd.cf.ac.uk/ac/introduction.php?lang=english), the single Nucleotide Polymorphism database (http://ftp.ncbi.nih.gov/snp/), genome aggregation database (gnomAD, https://gnomad.broadinstitute.org), 1000 genomes (https://www.internationalgenome.org/), Polyphen-2 (http://genetics.bwh.harvard.edu/pph2/), Mutation Taster (http://www.mutationtaster.org/), Sorting Intolerant from Tolerant (SIFT, https://sift.bii.a-star.edu.sg/) and Combined Annotation Dependent Depletion (CADD, https://cadd.gs.washington.edu/).
Wild-type human KIF21B cDNA (NCBI Reference Sequence: NM_001252100.1, https://www.ncbi.nlm.nih.gov/nuccore/NM_001252100.1)

Wild-type mouse Kif21b cDNA (NCBI Reference Sequence: NM_001039472.2, https://www.ncbi.nlm.nih.gov/gene/16565)
The raw data underlying Figs. 2a,b; 3b,d; 4b,d,e,g,h,j; 5b; 6c,d,e,g; 7c,d,e,g; and Supplementary Figs. 1d; 2d,f; 3a,b,d,f,h; 4a,b,d,f,g,h,i; 5a,d,g,j; 6c,d,h; 7b,c,d are provided in the Source Data file. All other relevant data included in the article are available from the authors upon request.
The three hKIF21B missense variants have been deposited in LOVD (Leiden Open Variation Database) v3.0 (https://databases.lovd.nl/shared/genes/KIF21B) under the accession number 0000663938 (p.Ile678Leu), 0000663939 (p.Gln313Lys) and 0000663940 (p.Ala1001Thr).

Field-specific reporting

Please select the one below that is the best fit for your research. If you are not sure, read the appropriate sections before making your selection.

☒ Life sciences ☐ Behavioural & social sciences ☐ Ecological, evolutionary & environmental sciences

For a reference copy of the document with all sections, see nature.com/documents/nr-reporting-summary-flat.pdf

Life sciences study design

All studies must disclose on these points even when the disclosure is negative.

Sample size	No statistical methods were used to pre-determine sample sizes but our sample sizes are similar to those reported previously (Broix, L. et al. Hum Mol Genet (2018) ; Van Riel, W.E. et al. Elife 6(2017) ; Kannan, M. et al. Proc Natl Acad Sci U S A (2017); Courchet, J. et al. Cell (2013)).
Data exclusions	No data was excluded from the analysis.
Replication	All the experimental findings were reliably reproduced at least three times and the total number of replicates (n) are indicated in the corresponding figure legends and in the statistic table (Supplementary Data 1).
Randomization	All WT animals were randomly assigned to experimental groups. All biological and biochemical experiments were carried out with appropriate internal negative and/or positive controls as indicated.
Blinding	Investigators were blinded for mouse in utero electroporation experiments only.

Reporting for specific materials, systems and methods

We require information from authors about some types of materials, experimental systems and methods used in many studies. Here, indicate whether each material, system or method listed is relevant to your study. If you are not sure if a list item applies to your research, read the appropriate section before selecting a response.

Materials & experimental systems

n/a	Involved in the study	
☐	☒	Antibodies
☐	☒	Eukaryotic cell lines
☒	☐	Palaeontology
☐	☒	Animals and other organisms
☐	☒	Human research participants
☒	☐	Clinical data

Methods

n/a	Involved in the study	
☒	☐	ChIP-seq
☒	☐	Flow cytometry
☐	☒	MRI-based neuroimaging

Antibodies

Antibodies used	β-III-tubulin (Eurogentec MMS-435P-0100); Caspase-3 (R and D system AF835); Cux1 (Proteintech HPA003317); CD24-APC Antibody (clone M1/69, ThermoFisher Scientific 17-0242-82); DIG antibody (Abcam ab76907); GFP (Abcam ab6673); GFP (Abcam GFP-1020); HA (Sigma-Aldrich 11867423001); Myc-Tag (Cell Signalling 71D10); KIF21B (Sigma-Aldrich HPA027274); KIF21B (Abcam ab135410); Pax6 (Biolegends 901301); Tau (Millipore MAB3420); Tbr2 (EBiosciences 14-4875-80); Actin coupled HRP (Sigma-Aldrich A3854); Goat-mouse-HRP (ThermoFisher Sc. G-21040); Goat-rabbit-HRP (ThermoFisher Sc. G-21234); Goat-rat-HRP (ThermoFisher Sc. 62-9520); Donkey-goat-488 (ThermoFisher Sc. A-11055); Donkey-mouse-488 (ThermoFisher Sc. A-21202); Donkey-mouse-555 (ThermoFisher Sc. A-31570); Donkey-rabbit-555 (ThermoFisher Sc. A-31572); Donkey-rabbit-488 (ThermoFisher Sc. R-37118); Donkey-rat-488 (thermoFisher Sc. A-21208); Ki67 coupled-570 (eBioscience 41 5698 80)
Validation	β-III-tubulin (https://www.biolegend.com/fr-fr/products/purified-anti-tubulin-beta-3-tubb3-antibody-11580); Caspase-3 (https://www.rndsystems.com/products/human-mouse-active-caspase-3-antibody_af835); Cux1 (https://www.atlasantibodies.com/products/antibodies/primary-antibodies/triple-a-polyclonals/cux1-antibody-hpa003317/); CD24-APC Antibody (https://www.thermofisher.com/antibody/product/CD24-Antibody-clone-M1-69-Monoclonal/17-0242-82); DIG antibody (https://www.abcam.com/digoxigenin-antibody-ab76907.html); GFP (https://www.abcam.com/gfp-antibody-ab6673.html); GFP (https://www.abcam.com/gfp-antibody-ab13970.html); HA (https://www.sigmaaldrich.com/catalog/product/roche/roahaha?lang=fr&region=FR); Myc-Tag (https://www.cellsignal.com/products/primary-antibodies/myc-tag-71d10-rabbit-mab/2278); KIF21B (https://www.sigmaaldrich.com/catalog/product/sigma/hpa027274?lang=fr&region=FR); KIF21B (https://www.abcam.com/kif21b-antibody-n-terminal-ab135410-protocols.html); Pax6 (https://www.biolegend.com/en-us/products/

purified-anti-pax-6-antibody-11511); Tau (https://www.merckmillipore.com/FR/fr/product/Anti-Tau-1-Antibody-clone-PC1C6,MM_NF-MAB3420); Tbr2 (https://www.thermofisher.com/antibody/product/14-4875-80.html?CID=AFLBC-14-4875-80); Actin coupled HRP (https://www.sigmaaldrich.com/catalog/product/sigma/a3854); Goat-mouse-HRP (https://www.thermofisher.com/antibody/product/Goat-anti-Mouse-IgG-H-L-Cross-Adsorbed-Secondary-Antibody-Polyclonal/G-21040); Goat-rabbit-HRP (https://www.thermofisher.com/antibody/product/Goat-anti-Rabbit-IgG-H-L-Cross-Adsorbed-Secondary-Antibody-Polyclonal/G-21234); Goat-rat-HRP (https://www.thermofisher.com/antibody/product/Goat-anti-Rat-IgG-H-L-Secondary-Antibody-Polyclonal/62-9520); Donkey-goat-488 (https://www.thermofisher.com/antibody/product/Donkey-anti-Goat-IgG-H-L-Cross-Adsorbed-Secondary-Antibody-Polyclonal/A-11055); Donkey-mouse-488 (https://www.thermofisher.com/antibody/product/Donkey-anti-Mouse-IgG-H-L-Highly-Cross-Adsorbed-Secondary-Antibody-Polyclonal/A-21202); Donkey-mouse-555 (https://www.thermofisher.com/antibody/product/Donkey-anti-Mouse-IgG-H-L-Highly-Cross-Adsorbed-Secondary-Antibody-Polyclonal/A-31570); Donkey-rabbit-555 (https://www.thermofisher.com/antibody/product/Donkey-anti-Rabbit-IgG-H-L-Highly-Cross-Adsorbed-Secondary-Antibody-Polyclonal/A-31572); Donkey-rabbit-488 (https://www.thermofisher.com/antibody/product/Donkey-anti-Rabbit-IgG-H-L-Secondary-Antibody-Polyclonal/R37118); Donkey-rat-488 (https://www.thermofisher.com/antibody/product/Donkey-anti-Rat-IgG-H-L-Highly-Cross-Adsorbed-Secondary-Antibody-Polyclonal/A-21208); Ki67 coupled-570 (https://www.thermofisher.com/antibody/product/Ki-67-Antibody-clone-SolA15-Monoclonal/41-5698-82)

Eukaryotic cell lines

Policy information about cell lines

Cell line source(s)	N2A, HEK 293T, COS7 and ST cells were provided by the cell culture platform of the IGBMC (Strasbourg).
Authentication	None of the cell lines used were authenticated.
Mycoplasma contamination	Cell lines used were tested for mycoplasm contamination (PCR test Venorgem) and confirmed mycoplasma free.
Commonly misidentified lines (See ICLAC register)	No commonly misidentified cell lines were used in the study.

Animals and other organisms

Policy information about studies involving animals; ARRIVE guidelines recommended for reporting animal research

Laboratory animals	Mice were bred at the IGBMC animal facility under controlled light/dark cycles, stable temperature (19°C) and humidity (50%) and were provided with food and water ad libitum. Timed-pregnant wild-type (WT) NMRI (Janvier-labs) and CD1 (Charles River Laboratories) females were used for in utero electroporation at embryonic day 14.5 (E14.5). Hybrid F1 females were obtained by mating inbred 129/SvJ females (Janvier-labs) with C57Bl/6J males (Charles River Laboratories). F1 females were crossed with C57Bl/6J males (Charles River Laboratories) and were used for in utero electroporation at E15.5. Electroporated embryos were collected at E16.5 or E18.5 and electroporated pups were collected two, four, eight or twenty-two days after birth (P2, P4, P8 or P22) after birth. E12.5 to E18.5 WT NMRI embryos (Janvier-labs) were used for Kif21b expression pattern experiments in Figure 2a-d and Suppl. Figure 2a-c. E16,5 mouse Rosa26-loxSTOP-YFP; NEXCRE/+ embryos were used for Fluorescent-activated cell sorting (FACS) in Suppl. Figure 2d. Kif21b mice were generated using the International Mouse Phenotyping Consortium targeting mutation strategy and obtained from UC Davis/ KOMP repository. Kif21b +/- females were crossed with Kif21b +/- males and P0 pups were used for immunofluoresence experiments in suppl. Figure 2e-f. WT E15.5 CD1 mouse embryos (Charles River Laboratories) were used for primary neuronal culture (Suppl. Figures 6 and 7) and WT E15.5 C57Bl/6J mouse embryos (Charles River Laboratories) were used for immunostaining in microchambers (Figure 2d).<br>Zebrafish (Danio rerio) embryos (AB strain) maintenance and experiments were performed as described here https://zfin.org/zf_info/zfbook/cont.html#cont1. Analysis were performed 1, 2, 4 or 5 days post-fertilization (dpf).
Wild animals	No wild animals were used.
Field-collected samples	No field samples were collected.
Ethics oversight	All animal studies were conducted in accordance with French regulations (EU Directive 86/609 – French Act Rural Code R 214-87 to 126) and all procedures were approved by the local ethics committee and the Research Ministry (APAFIS#15691-201806271458609).

Note that full information on the approval of the study protocol must also be provided in the manuscript.

Human research participants

Policy information about studies involving human research participants

Population characteristics	Patient 1 (NM_001252100.1, c.2032A>C, p.Ile678Leu) is the second child of healthy, Caucasian, non- consanguineous parents. He was born at full term with normal growth parameters and had an uncomplicated neonatal course. Concerns about development were raised at 18 months when he started walking. He had an uneven gait, stereotypies and no speech. Brain MRI at age two years revealed isolated complete agenesis of the corpus callosum (Fig. 1e). He presented with down-slanting palpebral fissures and downturned corners of the mouth. His first words were at 36 months. He currently has appropriate expressivelanguage despite persistent dysarthria. He was evaluated at six years and nine months (WISC IV) and total IQ was 78 which is consistent with borderline intellectual disability (ID). He has learning disabilities and is therefore cared for in a medico educational institution. He takes methylfenidate due to hyperactivity. Most recent neurological examination was unremarkable. The patient has muscle stiffness, but benefits from physical therapy to ease pain.

Patient 2 (NM_001252100.1, c.937C>A, p.Gln313Lys) is the child of healthy, non-consanguineous, African American parents. The pregnancy was complicated by intra uterine growth restriction and oligohydramnios. He was born at 38 weeks gestation with height at 49cm (49th percentile), weight at 2.584kg (7th percentile) and head circumference at 32cm (5th percentile). He had a nuchal cord at birth transient cyanosis associated with mild respiratory distress, but was discharged home without complications. He presented with severe developmental delay and. He was unable to sit and was non-verbal. Clinical examination at 12 years old showed growth difficulties with weight at 20kg (<1st percentile), height at 139 cm (8th percentile) and microcephaly with head circumference at 48.5cm (<1st percentile, -3.9 SD). He had poor visual fixation with constant tongue thrusting and poor head control. He presented with bilateral ankle tightness, and right wrist contracture.
Patient 3 (NM_001252100.1, c.3001G>A, p.Ala1001Thr) is a girl of non-consanguineous Dutch parents. Both parents have a reported personal history of mild intellectual disability. The father is a carrier of the variant and present with developmental delay and learning diffculties. The pregnancy was routine and she was born at full term with normal growth parameters. Her psychomotor development was delayed, she sat at ten months and walked at 24 months. She said her first words at 36 months of age. At age five years, she was able to speak in sentences. She was evaluated at nine years and verbal and performance IQ were 54 and 59 respectively, which is consistent with mild to moderate ID. Upon clinical examination, she had mild dysmoprhic features including epicanthal folds, mild ptosis, and tented upperlip. Her legs were mildly hypertonic. The brain MRI did not show any structural abnormalities.
Patient 4 (NM_001252100.1, c.2959_2962dup, p.Asn988Serfs*4) was born to non-canguinous parents. Mother had a seizure disorder and she took Topiramate during the pregnancy. Antenatal ultrasound was positive for the fetus measuring small for gestational age. He was born at 38 weeks gestation by cesarean section secondary to repeat maternal seizures. Neonatal mensurations confirmed hypotrophy with birth height at 43 cm (<1st percentile) and birth weight at 2633 g (8th percentile). He had feeding difficulties in the neonatal period requiring an NG tube. Problems persisted and he received a G-tube at 18 months. Currently, he takes mainly by mouth and uses the G-tube for medications. He has a history of moderate to severe constipation. At 37 months of age, he had a developmental quotient of 97 consistent with a history of mild global developmental delays. He was diagnosed with right Duane syndrome and has central sleep apnea requiring C pap after adenoidectomy. He had a chromosome microarray (CMA) that was non-diagnostic; it showed he was a carrier for Poretti-Boltshauser syndrome [OMIM: 615960] due to a single heterozygous LAMA1 deletion and a gain on 22q11.23 with no clinical onsequence associated. He has also had normal mitochondrial sequencing.

Recruitment

Patients were recruited through specialized genetics departments in France, Netherlands and USA. Patients were recruited with a prior diagnosis of intellectual diability, malformation of cortical development or agenesis of corpus callosum.

Ethics oversight

All parents signed an appropriate consent form for genetic analysis. Inclusion and genetic studies were approved by local ethics committee in France (CCP Ile de France, CPP N° 71-10/ ID RCB : 2010-A00802-37) and USA (Institutional Review Board at Baylor College of Medicine, protocol H-29697 and at the John Hopkins School of Medicine).

Note that full information on the approval of the study protocol must also be provided in the manuscript.

Magnetic resonance imaging

Experimental design

Design type

diagnostic MRI

Design specifications

No specific design.

Behavioral performance measures

No behavioral measurement were done

Acquisition

Imaging type(s)

structural

Field strength

1,5T

Sequence & imaging parameters

T1 sequence

Area of acquisition

Whole brain

Diffusion MRI ☐ Used ☒ Not used

Preprocessing

Preprocessing software

No pre-processing

Normalization

If data were normalized/standardized, describe the approach(es): specify linear or non-linear and define image types used for transformation OR indicate that data were not normalized and explain rationale for lack of normalization.

Normalization template

Describe the template used for normalization/transformation, specifying subject space or group standardized space (e.g. original Talairach, MNI305, ICBM152) OR indicate that the data were not normalized.

Noise and artifact removal

Describe your procedure(s) for artifact and structured noise removal, specifying motion parameters, tissue signals and physiological signals (heart rate, respiration).

Volume censoring | *Define your software and/or method and criteria for volume censoring, and state the extent of such censoring.*

Statistical modeling & inference

Model type and settings | No modeling in our study

Effect(s) tested | *Define precise effect in terms of the task or stimulus conditions instead of psychological concepts and indicate whether ANOVA or factorial designs were used.*

Specify type of analysis: ☐ Whole brain ☐ ROI-based ☐ Both

Statistic type for inference
(See Eklund et al. 2016) | *Specify voxel-wise or cluster-wise and report all relevant parameters for cluster-wise methods.*

Correction | *Describe the type of correction and how it is obtained for multiple comparisons (e.g. FWE, FDR, permutation or Monte Carlo).*

Models & analysis

n/a	Involved in the study
☒	☐ Functional and/or effective connectivity
☒	☐ Graph analysis
☒	☐ Multivariate modeling or predictive analysis
